# Chronic exposure to mining-contaminated borehole water impairs the entire male reproductive axis: partial mitigation by alpha-lipoic acid in Wistar rats

**DOI:** 10.3389/ftox.2026.1899884

**Published:** 2026-07-16

**Authors:** Festus Toochukwu Okposhi, Collins Nduka Esomchi, Kenneth Tochukwu Onwe, Leslie Ogechi Onumajuru, Ernest Maduabuchi Omeje, Favour Chioma Onwumere, Precious Chidiogo Ugwu, Goodness Emmanuella Onukogu, Jennifer Obianuju Olisaeke, Chinwendu Princess Victor, Chiemerie Promise Omeh, Bianca Adanna Onele

**Affiliations:** 1 Department of Anatomy, Faculty of Basic Medical Sciences, Alex Ekwueme Federal University Ndufu Alike Ikwo, Ebonyi State, Nigeria; 2 Department of Human Anatomy, Faculty of Basic Medical Sciences, State University of Medical and Applied Sciences, Igbo Eno, Enugu State, Nigeria

**Keywords:** alpha-lipoic acid, borehole water, heavy metal contamination, HPG axis, male reproductive toxicity, oxidative stress

## Abstract

**Introduction:**

Mining activities, industrial discharges, and poor waste management in many developing countries, including Nigeria, have led to widespread contamination of groundwater with mixtures of heavy metals such as lead, cadmium, arsenic, and mercury. In mining-impacted regions of Ebonyi State, borehole water, the primary drinking source for rural communities regularly exceeds WHO permissible limits, resulting in chronic low-dose exposure that threatens male reproductive health through disruption of the entire hypothalamus–pituitary–gonadal (HPG) axis. This study investigated the reproductive toxicity of naturally contaminated borehole water and evaluated the protective and therapeutic potential of alpha-lipoic acid (ALA) in adult male Wistar rats.

**Methodology:**

Thirty adult male Wistar rats were divided into five groups (n = 5): normal control, heavy metal-contaminated water only (28 days), ALA only (25 mg/kg), post-treatment (contaminated water 28 days followed by ALA 14 days), and pre-treatment (ALA 14 days followed by contaminated water 28 days). Water from a mining area in Ebonyi State, Nigeria, contained Pb (2.4 mg/L), Cd (1.7 mg/L), As (1.2 mg/L), and Hg (0.8 mg/L), exceeding WHO limits. After sacrifice, serum hormones (GnRH, FSH, LH, testosterone), antioxidant status, inflammatory markers, sperm parameters, steroidogenic enzymes, and histopathology of the hypothalamus, pituitary, and testes were assessed.

**Results:**

Contaminated water significantly reduced body weight, organ weights, sperm quality, GnRH, FSH, testosterone, antioxidant enzymes, and steroidogenic protein expression while increasing inflammation and histopathological damage across the HPG axis. Post-treatment with ALA significantly improved most parameters compared to the heavy metal-only group, whereas pre-treatment offered limited protection. However, neither regimen fully restored values to control levels.

**Conclusion:**

Chronic exposure to naturally heavy metal-contaminated drinking water induces profound multi-level HPG axis dysfunction. Alpha-lipoic acid at 25 mg/kg provides significant but incomplete protection, with post-exposure administration being more effective. These findings highlight the need for higher doses or combination therapies and underscore the urgent public health risk in affected regions.

## 1 Introduction

Every environment on Earth, whether urban, industrial, or agricultural, faces unique ecological and health challenges, with environmental toxicity being a significant concern (Nandomah and Tetteh, 2024). Industrial expansion, urbanization, and poor waste management have contaminated ecosystems, posing risks to both wildlife and humans (Rimi Abubakar et al., 2022). Heavy metal pollution, including non-biodegradable elements like lead, cadmium, arsenic, mercury, chromium, and nickel, is particularly alarming (Laoy et al., 2025). These metals are released through various activities and can persist in the environment, undergoing cycles that allow them to travel long distances, even reaching remote areas (Oladimeji et al., 2024).

The environment is the primary provider of the two most fundamental human needs: food and water (Rojas et al., 2023). Crops absorb heavy metals from contaminated soil and water, while aquatic organisms accumulate them in their tissues. Livestock grazing on polluted pastures also incorporate these metals into meat and milk, and groundwater serves as a direct source of chronic exposure for over two billion people globally (Laoy et al., 2025). In many developing regions, unregulated industrial discharges and poor water treatment have led to heavy metal concentrations in drinking water that exceed WHO and EPA limits, creating a public health crisis (Okareh et al., 2023; Ugwu et al., 2022).

Heavy metals, once ingested or inhaled, are distributed through the bloodstream and cause multi-organ toxicity via various mechanisms (Yang et al., 2025). They catalyze Fenton and Haber–Weiss reactions, generating reactive oxygen species (ROS); deplete and inactivate antioxidants like glutathione (Uchewa and Ezugworie, 2019); bind to critical enzymes and proteins; and substitute for essential metals in metalloproteins, disrupting cellular balance. This leads to oxidative stress, chronic inflammation, DNA damage, mitochondrial dysfunction, and premature cellular aging across multiple physiological systems (Famurewa et al., 2023; Uchewa et al., 2022).

The male reproductive system is highly susceptible to heavy metal toxicity due to its effects on the hypothalamus–pituitary–gonadal (HPG) axis (Marinaro et al., 2025), which regulates reproductive functions through hormones like GnRH, LH, FSH, and testosterone (Oduwole et al., 2021). Heavy metals can cross the blood–brain barrier, impairing GnRH production and secretion, altering pituitary responsiveness, damaging Leydig and Sertoli cells, and disrupting key steroid synthesis enzymes (Famurewa et al., 2023; Santiago-Andres et al., 2025). This leads to germ cell apoptosis and sperm DNA fragmentation, resulting in reduced testosterone levels, oligospermia, asthenozoospermia, teratozoospermia, and increased infertility rates, particularly noted in workers exposed to heavy metals and communities near contaminated water sources (Inhorn et al., 2008; Marić et al., 2021).

Oxidative stress plays a central role in heavy-metal toxicity, leading to increased interest in compounds that can mitigate these effects (Uchewa et al., 2022). Traditional treatments, such as chelating agents (e.g., CaNa_2_-EDTA, DMSA), have been the standard for managing heavy metal poisoning for years (Kim et al., 2019). While effective in mobilizing metals and promoting urinary excretion, they have significant drawbacks: limited ability to penetrate the central nervous system and testes, potential redistribution of metals, depletion of essential trace elements, nephrotoxicity (Balali-Mood et al., 2025), and failure to reverse oxidative damage in the HPG axis (Rachamalla et al., 2022). These limitations have prompted research into alternative therapies that can neutralize ROS, repair cellular damage, and restore endocrine function.

Antioxidant-based therapies are becoming popular as safe and effective methods to reduce heavy metal-induced reproductive toxicity. One key antioxidant, alpha-lipoic acid (ALA), is unique because it is both water- and lipid-soluble, allowing it to act in various cellular environments (Famurewa et al., 2023). ALA scavenges harmful radicals, regenerates essential antioxidants like glutathione and vitamin C, enhances detoxification enzyme expression, chelates transition metals, and has anti-inflammatory effects inhibition (Famurewa et al., 2023; Superti and Russo, 2024). Significantly, ALA crosses the blood-brain barrier and accumulates in hypothalamic and pituitary tissues, showing protective effects against heavy metal reprotoxicity (Famurewa et al., 2023; Maciejczyk et al., 2022).

However, most previous studies have used single heavy metals or artificially prepared mixtures administered by gavage, which do not accurately reflect real-world environmental exposure through drinking water. To our knowledge, few studies have examined the effects of naturally contaminated borehole water on the complete HPG axis or evaluated the timing-dependent (pre-versus post-exposure) protective effects of ALA.

Therefore, the present study investigated the reproductive toxicity of naturally heavy metal-contaminated borehole water collected from a mining-impacted area in Ebonyi State, Nigeria, and evaluated the ameliorative potential of oral alpha-lipoic acid (25 mg/kg) administered either before or after exposure in adult male Wistar rats.

## 2 Materials and methods

### 2.1 Ethical approval

Approval for ethical considerations in this research was secured from the Ethical Committee of the Faculty of Basic Medical Sciences at Alex Ekwueme Federal University, Ndufu Alike, Ikwo (AE-FUNAI), located in Ebonyi State, Nigeria, in accordance with institutional protocols for animal studies, bearing the reference number AE-FUNAI/FBMS/EAHC/26/007. All procedures complied with the institutional guidelines of AE-FUNAI and were conducted in accordance with the ARRIVE 2.0 guidelines for reporting animal research (Percie du Sert et al., 2020), the NIH Guide for the Care and Use of Laboratory Animals (8th edition) (National Research Council, 2011), and OECD principles.

### 2.2 Randomization and blinding

Animals were randomly assigned to the five experimental groups using a computer-generated randomization sequence (GraphPad QuickCalcs). All personnel involved in data collection, tissue processing, and histopathological/histomorphometric scoring were blinded to group allocation. Code breaking was performed only after statistical analysis was completed.

### 2.3 Sample size justification

The sample size of n = 5 per group was determined based on previous similar reproductive toxicity studies using heavy metals and antioxidants in Wistar rats, which typically detect statistically significant differences with this group size (Oyeyemi et al., 2022). Power analysis (using G*Power software, effect size f = 0.8, α = 0.05, power = 0.80) indicated that n = 5 per group was sufficient for the primary endpoints (testosterone, sperm count, and Johnsen’s score).

### 2.4 ALA purchase and preparation

Alpha-lipoic acid was procured from Sigma-Aldrich (United States). The compound was stored according to the manufacturer’s instructions and reconstituted in an appropriate vehicle (corn oil) before administration. All other reagents were of analytical grade. The dose of ALA was dissolved in 2 mL/kg (body weight) of corn oil. The ALA dose (25 mg/kg b. w./day) used in the current study was adopted from a previous study by Famurewa et al. (2023).

### 2.5 Water collection for heavy metal exposure

Water samples containing heavy metals were collected from a drinking borehole in Alike, Ikwo Local Government Area of Ebonyi State. The sampling site is located near the Royal Salt mining area, which is known to contribute to elevated heavy metal levels in surrounding soil and water bodies (Okafor et al., 2024; Okafor, 2025). The water samples were filtered and analysed using atomic absorption spectrophotometry following the methods of Magaji et al. (2023) to quantify concentrations of key heavy metals such as lead (Pb), cadmium (Cd), mercury (Hg), arsenic (As) and other metals. The instrument parameters were set according to the manufacturer’s specifications, and each sample was aspirated into the flame for measurement as reported by Orisakwe et al. (2017). Three replicate readings were taken for each metal to ensure accuracy, and the mean concentration was calculated from the calibration curve. The detected metal concentrations in the water samples were compared to the World Health Organisation (WHO) permissible limits to assess the level of contamination. In addition to heavy metal analysis, key physicochemical parameters of the borehole water were determined.

### 2.6 Experimental design

Thirty (30) adult male Wistar rats weighing 130–180 g were obtained from the Animal House of Alex Ekwueme Federal University. The rats were housed in well-ventilated cages at room temperature (22 °C–25 °C) under a 12 h light/dark cycle and provided with standard feed and water *ad libitum*. Animals were acclimatised for 2 weeks before the commencement of the experiment. The animals were randomly assigned to five groups (n = 5 per group). The total study duration was 42 days for all groups to ensure proper time-matching.

- Group A (Control): received an equivalent volume of distilled water (1 mL/kg) by oral gavage daily for 42 days.
- Group B (Heavy Metal + Recovery): Exposed to heavy metal-contaminated water for 28 days, followed by distilled water for another 14 days.
- Group C (ALA Only): Administered ALA at 25 mg/kg b. w/day orally for 14 days.
- Group D (Post-Treatment): Exposed to heavy metal-contaminated water for 28 days, followed by ALA treatment for 14 days.
- Group E (Pre-Treatment): Pre-treated with ALA for 14 days, followed by heavy metal exposure for another 28 days.

**FIGURE 1:**
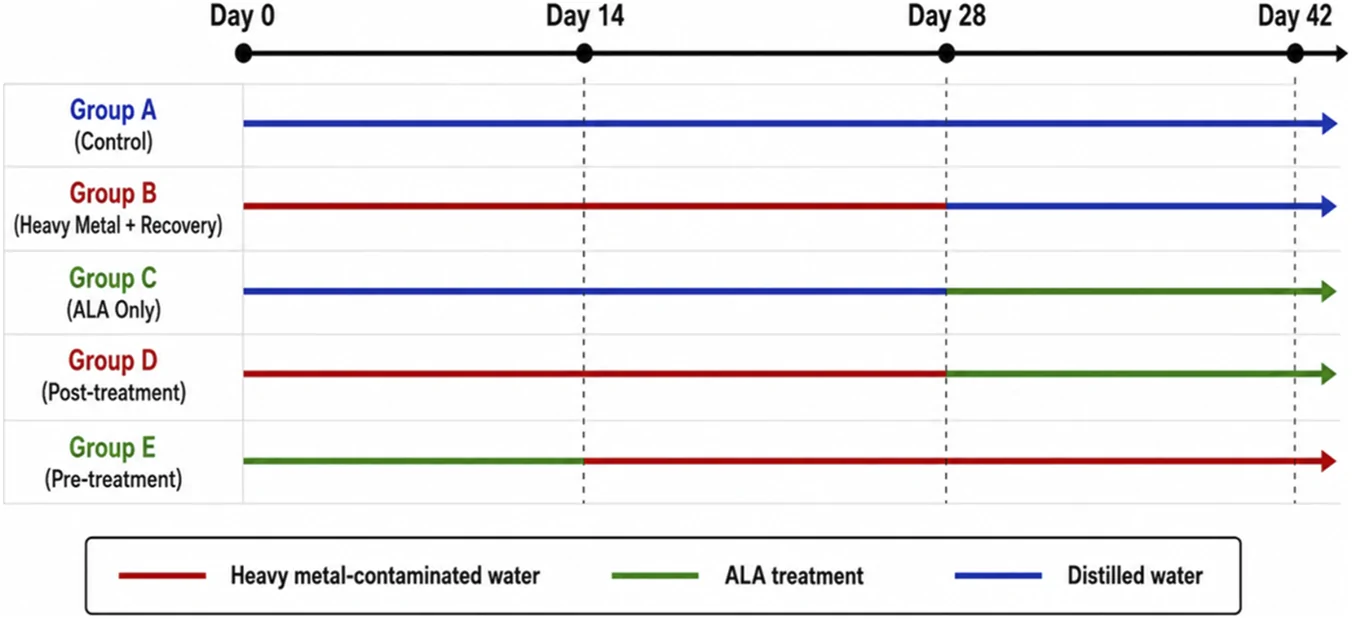
Schematic representation of the experimental timeline showing the treatment schedule for all five groups over the 42-day study period.

A schematic diagram of the experimental timeline is presented in Figure 1.

A 28-day exposure period was chosen because it is widely used in sub-chronic heavy metal toxicity studies (Nasiadek et al., 2018; Adelakun et al., 2020) and reflects the realistic duration of continuous exposure to contaminated borehole water in affected communities.

All treatments were administered by oral gavage once daily between 07:00 and 08:00 h using a curved feeding needle. Body weights were recorded weekly.

### 2.7 Organs collection and sample preparation

At the end of the experiment, animals were sacrificed via cervical dislocation under anaesthesia using intraperitoneal injection of ketamine (50 mg/kg) and xylazine (10 mg/kg). Blood samples were collected by heart puncture and centrifuged (3000 rpm for 15 min) to obtain sera, and they were then separated and stored at −80 °C for biochemical and hormonal assays.

After blood collection (within 5 min of euthanasia), the brain and testes were removed and weighed. The hypothalamus, pituitary gland, and left testis were fixed in 10% neutral buffered formalin (1:20 tissue-to-fixative ratio). To ensure proper fixative penetration, the testes were punctured with a fine needle before immersion. Tissues were fixed for 24–48 h at room temperature.

For Epididymal fluid collection, the caudal epididymis was cleaned and placed in a pre-warmed Petri dish with 1 mL of 0.9% saline at 37 °C. A small incision was made, and gentle pressure was applied to extrude the contents. The sperm suspension was incubated for 5 min at 37 °C before analysis.

### 2.8 Relative brain size, gonadosomatic index (GSI) and testicular volume

Relative brain size was determined by expressing brain weight as a percentage of body weight:

$$\text{Relative brain size }\left(\%\right)=\frac{\text{Brain}\;\text{weight}\;}{\text{Body}\;\text{weight}\;}\;X\;100$$

Similarly, the gonadosomatic index (GSI) was calculated to evaluate the relative size of the testes in relation to body weight using the formula previously reported by Esomchi et al. (2025):

$$\text{GSI }\left(\%\right)=\frac{\text{Testis}\;\text{weight}\;}{\text{Body}\;\text{weight}\;}\;X\;100$$

To assess testis volume, a sliding digital Vernier calliper was utilised to gauge the length and width of each testis. The volume of the testis was subsequently computed using the spheroid formula documented earlier by Esomchi et al. (2025):

$${Testes\;volume=Width}^{2}\;x\;length\;x\;0.523\;\left({mm}^{3}\right)$$

### 2.9 Semen analysis

Sperm motility, count, and morphology were evaluated following Famurewa et al. (2023). Sperm count was performed with a Neubauer hemocytometer, motility was assessed by light microscopy at ×40 magnification, and morphology was examined using eosin-nigrosin staining under ×100 oil immersion.

### 2.10 Biochemical assay

- Estimation of Heavy Metals in Serum: Heavy metal levels were assessed using an AAS-based method modified from a previously reported method by Famurewa et al. (2023). One millilitre of each serum was digested with 2.0 mL concentrated nitric acid for 20 h, followed by 0.5 mL of 72% perchloric acid and heating at 100 °C for 3 h, with an additional 4-h digestion. The cooled digest was topped up to 3.0 mL with deionised water, evaporated to dryness, and the residue reconstituted. Concentrations of Pb, Cd, Hg, and other metals were then measured using Atomic Absorption Spectrophotometry equipped with an EA3 furnace.
- Antioxidant markers: Catalase (CAT) activity was determined using the spectrophotometric method of Aebi (1984). Reduced glutathione (GSH) was quantified by the method of Güntherberg and Rost (1966).
- Hormonal assays: Serum levels of GnRH, LH, FSH, and testosterone were measured using commercially available rat-specific ELISA kits (catalog numbers: GnRH–E-EL-R0393; LH–E-EL-R0026; FSH–E-EL-R0391; Testosterone–E-EL-R0389; Elabscience, United States). The sensitivity of the assays was 9.38 pg/mL (GnRH), 0.19 mIU/mL (LH), 0.23 mIU/mL (FSH), and 0.06 ng/mL (testosterone), respectively. All samples were analysed in duplicate according to the manufacturer’s instructions.
- Expression of steroidogenesis: Steroidogenic enzyme activities of 3β-HSD, StAR and 17β-HSD were assessed using Novex Rat ELISAKit (Invitrogen, Camarillo, CA, United States).
- Inflammatory parameters*:* The testis homogenate was employed to determine the testis inflammatory parameters, including Interleukin-4 (IL-4) and tumour necrosis factor-alpha (TNF-α). Commercially available ELISA assays were used to measure the levels of these inflammatory factors, following the instructions provided by the manufacturer.
- Apoptotic caspase activity: Serum caspase 3 and 9 were determined with rat ELISA kits following the manufacturer’s directions.

### 2.11 Histology analysis

The hypothalamus, pituitary, and left testis tissues were preserved in 10% neutral-buffered formalin for 24–48 h to maintain their structural integrity. After fixation, the tissues underwent dehydration through increasing concentrations of ethanol (70%, 80%, 90%, 95%, and absolute alcohol), were cleared with two changes of xylene, and were then embedded in molten paraffin wax heated to 56 °C–58 °C. Once the paraffin blocks had solidified, they were sectioned at 5 µm thickness using a rotary microtome (Leica RM2235, Germany). The sections were floated on a warm water bath, placed on clean glass slides treated with Mayer’s albumin, and then dried at 37 °C.

Routine haematoxylin and eosin (H&E) staining was utilised. In brief, sections were deparaffinized using xylene, rehydrated through a series of decreasing alcohol concentrations, and rinsed with distilled water. Slides were soaked in Harris haematoxylin for 5 min, washed under running tap water, briefly differentiated in 1% acid alcohol, and then blued in alkaline water. Eosin was used for counterstaining for 2 min, after which slides were dehydrated in ascending concentrations of alcohol, cleared in xylene, and mounted with DPX mountant as previously described by Esomchi et al. (2026).

All histological samples were analysed using a light microscope (Olympus CX43, Japan) at a magnification of ×400. Photographs were taken with an Olympus EP50 digital camera for record-keeping. Structural and cellular alterations were assessed qualitatively by comparing them with the control group.

### 2.12 Testicular histomorphometry

Histomorphometric analyses were performed on tissues from all five animals per group (n = 5). For each animal, four paraffin sections (5 μm) were prepared, and six microscopic fields were analysed per section, giving a total of 24 fields per animal. All measurements were conducted by two independent blinded observers using Fiji (ImageJ) software.

#### 2.12.1 Testicular Component Area Fractions

The measured testicular components included the seminiferous epithelium, interstitial space, connective tissue, and lumen. These were assessed using the Fiji 84-intersection grid. The grid was applied to H&E-stained images captured at ×100 magnification, and the number of grid points intersecting each component was counted. For each animal, the mean number of intersecting points for each testicular component was determined from 24 camera fields, and the area fractions were then calculated using the following formula (Kangawa et al., 2019):

$$\text{Area fraction}=\frac{\left(\text{area}\;\text{per}\;\text{point}\times \text{average}\;\text{number}\;\text{of}\;\text{intersecting}\;\text{points}\right)}{\text{total}\;\text{area}\;\text{of}\;\text{photomicrograph}}\;X\;100\%$$

#### 2.12.2 Seminiferous tubule morphometry

The dimensions, diameter of the tubules, and the height of the epithelium in the seminiferous tubules were assessed in 50 round or nearly round tubules for each animal using Fiji software. In the seminiferous tubules, the cross-sectional area was established by tracing their perimeter with the tool, the freehand selection in Fiji. The mean diameter of the tubules and epithelial height were determined from the measurements of both the minor and major axes (Kumar and Nagar, 2014). In order to eliminate longitudinal tubules, only the mean tubule diameter was taken into account when D1/D2 was greater than or equal to 0.85; a D1/D2 ratio of 1.0 represented a perfect circle (Olasile et al., 2018).

#### 2.12.3 Germinal epithelium cell quantification

The amounts of different germinal epithelium cells, which include the Sertoli cells, spermatocytes, spermatogonia, and spermatids (both round and elongated), were counted in H&E-stained sections observed at ×400 magnification. For each animal, about 10 rounded VII seminiferous tubules were examined (Qiu et al., 2013).

#### 2.12.4 Leydig cell morphometry

The diameters of 50 nuclei, each with distinct nucleoli, were measured for each animal and analysed with Fiji software across all groups, based on H&E-stained images taken at ×400 magnification. The nucleus volume was calculated using the formula provided below (M et al., 2012).

$$Leydig\;cell\;nuclear\;volume=\frac{4}{3}{\pi R}^{3}\;\left({µm}^{3}\right)$$

where Radius (R) = D/2 and π = 3.14.

### 2.13 Histopathological evaluation

#### 2.13.1 Johnsen’s testicular score

Spermatogenesis was assessed using Johnsen’s testicular scoring system in 50 seminiferous tubules (stages II–VII) per animal, giving a total of 300 tubules per group. Each tubule was graded on a scale of 10 to 1 according to the modified Johnsen criteria:

- 10**:** Numerous spermatozoa present in the lumen.
- 9: Many elongated spermatids observed in the apical region of the epithelium.
- 8: A large number of elongated spermatids still attached to the Sertoli cell membrane.
- 7: Few elongated spermatids but many round spermatids.
- 6: No elongated spermatids, with only a few round spermatids.
- 5: No spermatids but a substantial number of spermatocytes.
- 4: Only a small number of spermatocytes.
- 3: Presence of spermatogonia cells only.
- 2: Only Sertoli cells present.
- 1: Absence of seminiferous epithelial cells.

The number of tubules corresponding to each score was multiplied by its respective score value. The sum of these products was then divided by 50 (the total number of tubules assessed) to obtain the mean Johnsen’s score for each group, which was used for further analysis (Owembabazi et al., 2023).

#### 2.13.2 Histological changes

Histological alterations in H&E-stained hypothalamus, pituitary gland and testicular sections were evaluated across 24 microscopic fields per animal, giving a total of 144 fields per group. The observed changes were graded semiquantitatively on a five-point scale as follows:

- **Grade 4:** Very severe; alterations present in more than 75% of the field.
- **Grade 3:** Severe; changes affecting 50%–75% of the field.
- **Grade 2:** Moderate; alterations observed in more than 25% but less than 50% of the field.
- **Grade 1:** Mild; changes visible in less than 25% of the field.
- **Grade 0:** No change; no observable alterations in the field.

This grading system followed the method previously described by Erpek et al. (2007). Histological slides were evaluated by two independent observers who were blinded to the experimental groups. Inter-observer variability was resolved by consensus.

### 2.14 Statistical analysis

Data were expressed as mean ± standard deviation (SD). One-way analysis of variance (ANOVA) followed by Tukey’s *post hoc* test was used for multiple comparisons. For non-parametric data (Johnsen’s score and histological grading), the Kruskal–Wallis test was utilised, followed by Dunn’s multiple comparisons test. All analyses were conducted using GraphPad Prism version 8 (GraphPad Software, San Diego, United States). Statistical significance was considered at p < 0.05.

## 3 Results

### 3.1 Water sample analysis

As shown in Table 1, the mean concentrations of Pb (2.4 mg/L), Cd (1.7 mg/L), Cr (2.9 mg/L), As (1.2 mg/L), Hg (0.8 mg/L), Fe (3.7 mg/L), Zn (6.03 mg/L), Cu (4.16 mg/L), Mn (1.3 mg/L), and Ni (1.9 mg/L) were recorded in the water sample. When compared with the WHO permissible limits of 0.01, 0.003, 0.05, 0.01, 0.006, 0.3, 3.0, 2.0, 0.1, and 0.07 mg/L, respectively, all the detected heavy metals exceeded the recommended limits, with Pb, Cd, As, and Hg showing markedly higher values relative to safe standards. This indicates that the water sample is contaminated with multiple heavy metals at levels above WHO safety thresholds.

**TABLE 1:** Heavy metal concentrations in water sample.

Heavy metal	WHO limit (mg/L)	Reading 1 (mg/L)	Reading 2 (mg/L)	Reading 3 (mg/L)	Mean (mg/L)
Pb (Lead)	0.01	2.2	2.5	2.4	2.4
Cd (Cadmium)	0.003	1.6	1.8	1.7	1.7
Cr (Chromium)	0.05	2.8	3.0	2.9	2.9
As (Arsenic)	0.01	1.1	1.3	1.2	1.2
Hg (Mercury)	0.006	0.7	0.9	0.8	0.8
Fe (Iron)	0.3	3	3.2	3.8	3.7
Zn (Zinc)	3.0	6	6.1	6	6.03
Cu (Copper)	2.0	4	4	4.5	4.16
Mn (Manganese)	0.1	1.2	1.4	1.3	1.3
Ni (Nickel)	0.07	1.8	2.0	1.9	1.9

The contaminated borehole water also exhibited acidic pH (5.4), elevated electrical conductivity (175 μS/cm), total hardness (41 mg/L), and turbidity (41 NTU) as presented in Table 2.

**TABLE 2:** Physicochemical properties of the heavy metal-contaminated borehole water.

Parameters	Values
Temperature (°C)	30
pH	5.4
Electrical Conductivity (EC) (µS/cm)	175
Total dissolved solids (TDS) (mg/L)	115
Total suspended solids (TSS) (mg/L)	14
Free alkalinity (mg/L)	0.12
Dissolved oxygen (DO) (mg/L)	24
Turbidity (NTU)	41
Total hardness (mg/L)	41
Colour (Pt-Co)	9
Chloride (Cl^−^) (mg/L)	50
Nitrate (NO_3_^−^) (mg/L)	24
Biochemical oxygen demand (BOD) (mg/L)	15
Chemical oxygen demand (mg/L)	33

### 3.2 Body morphometry

Body weight changes across experimental groups are shown in Table 3. Group A (control) showed a normal weight gain of 33.50 g. Exposure to heavy-metal-contaminated water (Group B) caused a significant weight reduction (−31.25 g) compared to Group A. Alpha-lipoic acid alone (Group C) produced the highest weight gain, which was significantly greater than Group B. Post-treatment with ALA after heavy-metal exposure (Group D) resulted in moderate weight gain (15.75 g), significantly different from both Groups B and C. Pre-treatment with ALA before heavy-metal exposure (Group E) showed a slight weight reduction (−3.25 g), which was significantly different from Groups A, B, and C.

**TABLE 3:** Body weight changes in experimental groups.

Groups	Initial weight (g)	Final weight (g)	Weight change (g)	Weight gain (%)
A	159.00 ± 3.74	192.50 ± 15.00	33.50 ± 15.46	21.12 ± 9.80
B	174.00 ± 1.41	142.80 ± 2.75	−31.25 ± 4.11^a^	−17.95 ± 2.23^a^
C	135.30 ± 7.41	184.80 ± 18.57	49.50 ± 8.17^b^	36.65 ± 11.98^b^
D	149.30 ± 3.40	165.00 ± 17.32	15.75 ± 15.54^bc^	10.48 ± 10.35^bc^
E	150.30 ± 4.03	147.00 ± 4.76	−3.25 ± 8.22^ac^	−2.07 ± 2.68^abc^

Values represent Mean ± SD. a represents a significant difference when compared to Group A. b represents a significant difference when compared to Group B. c represents a significant difference when compared to Group C.

Brain and testicular morphometry across experimental groups are shown in Table 4. Control had a brain weight of 1.86 g and testes weight of 1.37 g. Heavy-metal exposure in Group B caused a significant reduction in brain weight and testes weight compared to Group A. Alpha-lipoic acid alone (Group C) produced the highest brain weight (2.20 g) and testes weight (1.55 g), significantly greater than Group B. Post-treatment with ALA following heavy-metal exposure (Group D) resulted in brain and testes weights that were significantly different from Groups B and C. Pre-treatment with ALA showed intermediate values, with testes and brain weights significantly different from Groups A and C and brain weight also significantly different from Group B. Testes volume followed a similar pattern, with Group C recording the highest volume (1.29 mm) and Group B the lowest (0.78 mm).

**TABLE 4:** Brain and testicular morphometry.

Groups	Brain weight (g)	Brain-body weight ratio (%)	Testes weight (g)	GSI (%)	Testes volume (mm^3^)
A	1.86 ± 0.01	1.01 ± 0.01	1.37 ± 0.05	0.74 ± 0.03	1.07 ± 0.18
B	1.23 ± 0.01^a^	0.86 ± 0.01	1.20 ± 0.01^a^	0.85 ± 0.02	0.78 ± 0.03
C	2.20 ± 0.14^ab^	1.29 ± 0.16^ab^	1.55 ± 0.06^ab^	0.91 ± 0.09	1.29 ± 0.02^b^
D	1.59 ± 0.02^abc^	0.88 ± 0.01^c^	1.37 ± 0.03^bc^	0.76 ± 0.01	1.00 ± 0.01
E	1.39 ± 0.01^ac^	0.93 ± 0.00^c^	1.25 ± 0.01^c^	0.84 ± 0.01	0.97 ± 0.04

Values represent Mean ± SD. a represents a significant difference when compared to Group A; b represents a significant difference when compared to Group B; c represents a significant difference when compared to Group C.

### 3.3 Semen analysis

Effects of ALA and heavy-metal exposure on semen parameters are presented in Table 5. Sperm motility was highest in Group C (75%), significantly greater than Group B, while Group B showed the lowest active motility and highest non-motile sperm (30%) compared to the control. Post-treatment with ALA (Group D) resulted in moderate motility (60%), higher than Group B but lower than Group C, whereas pre-treatment (Group E) showed motility of 55%. Sperm count followed a similar trend, with Group C recording the highest concentration (40 × 10^6^/mL), significantly greater than Group B (14 × 10^6^/mL), and Groups D and E showing intermediate values. Normal sperm morphology was highest in Group C (91%), significantly different from Group B (77%), while Groups D and E had slightly lower percentages (85.5% and 83.5%). Among defects, pinhead abnormalities were notably lower in Group C (2.5%) compared to Group B (6.5%). Other head, midpiece, and tail defects were generally low across all groups.

**TABLE 5:** Effects of ALA and HMW on the semen analysis.

Groups	A	B	C	D	E
Motility (%)					
Active motile	65.00 ± 7.07	35.00 ± 7.07^a^	75.00 ± 7.07^b^	60.00 ± 0.00	55.00 ± 7.07
Sluggish motile	25.00 ± 7.07	35.00 ± 7.07	15.00 ± 7.07	25.00 ± 7.07	30.00 ± 0.00
Non-motile	10.00 ± 0.00	30.00 ± 0.00^a^	10.00 ± 0.00^b^	15.00 ± 7.07	15.00 ± 7.07
Count (106/mL)					
​	26.50 ± 3.54	14.00 ± 1.41^a^	40.00 ± 1.41^ab^	22.00 ± 2.83^c^	19.00 ± 1.41c
Morphology (%)					
Normal sperm	87.00 ± 1.41	77.00 ± 1.41^a^	91.00 ± 2.83^a^	85.50 ± 3.54	83.50 ± 2.12
Head defects (%)					
Round head	0	0	0	0	0
Pinhead	3.50 ± 0.71	6.50 ± 0.71	2.50 ± 0.71^b^	3.50 ± 0.71	4.00 ± 1.41
Midpiece defects (%)					
Bent midpiece	2.00 ± 0.00	5.00 ± 2.83	2.00 ± 0.00	3.50 ± 0.71	4.00 ± 0.00
Coiled midpiece	0	0	0	0	0
Tail defects (%)					
Headless tail	4.50 ± 0.71	6.00 ± 0.00	2.50 ± 0.71	4.00 ± 1.41	4.50 ± 0.71
Coiled tail	0	0	0	0	0
Absence of tail	0	0	0	0	0
Loop tail	1.00 ± 0.00	2.50 ± 0.71	0.50 ± 0.71	1.00 ± 0.00	1.50 ± 0.71

Values represent Mean ± SD. a represents a significant difference when compared to Group A; b represents a significant difference when compared to Group B; c represents a significant difference when compared to Group C.

### 3.4 Biochemical assay

#### 3.4.1 Estimation of heavy metals in serum

Serum concentrations of heavy metals across groups are shown in Table 6. Animals exposed to contaminated water (Group B) exhibited markedly elevated levels of all measured metals compared with the control group. ALA alone (Group C) showed the lowest concentrations across all parameters and differed significantly from Group B. Post-treatment (Group D) reduced metal levels relative to Group B but remained significantly higher than those of Group C. Pre-treatment (Group E) also demonstrated partial reductions, although values generally remained elevated and significantly different from Groups C and D in most comparisons.

**TABLE 6:** Serum concentrations of heavy metals across groups.

Heavy metal	Group A	Group B	Group C	Group D	Group E
Pb (mg/L)	0.14 ± 0.04	2.30 ± 0.14^a^	0.04 ± 0.02^b^	1.50 ± 0.14^abc^	2.05 ± 0.07^acd^
Cd (mg/L)	0.11 ± 0.02	1.50 ± 0.14^a^	0.03 ± 0.02^b^	0.95 ± 0.07^abc^	1.10 ± 0.14^abc^
Cr (mg/L)	0.48 ± 0.04	2.70 ± 0.14^a^	0.15 ± 0.06^b^	1.65 ± 0.07^abc^	2.10 ± 0.14^abcd^
As (mg/L)	0.23 ± 0.03	1.05 ± 0.07^a^	0.19 ± 0.02^b^	0.30 ± 0.14^abc^	0.75 ± 0.07^acd^
Hg (mg/L)	0.28 ± 0.03	1.60 ± 0.00^a^	0.80 ± 0.03^b^	0.95 ± 0.07^abc^	1.35 ± 0.07^abcd^
Fe (mg/L)	0.98 ± 0.11	4.10 ± 0.28^a^	0.33 ± 0.04^b^	2.70 ± 0.28^abc^	2.85 ± 0.21^abcd^
Zn (mg/L)	1.25 ± 0.07	4.65 ± 0.50^a^	0.56 ± 0.09^b^	2.60 ± 0.42^abc^	3.30 ± 0.42^abcd^
Cu (mg/L)	0.95 ± 0.07	5.65 ± 0.21^a^	0.40 ± 0.28^b^	3.55 ± 0.21^abc^	4.45 ± 0.21^abcd^
Mn (mg/L)	0.81 ± 0.04	2.10 ± 0.14^a^	0.17 ± 0.06^ab^	1.15 ± 0.21^bc^	1.70 ± 0.14^acd^
Ni (mg/L)	0.12 ± 0.02	1.85 ± 0.07^a^	0.04 ± 0.01^b^	0.95 ± 0.07^abc^	1.30 ± 0.00^abcd^

Values represent Mean ± SD. a represents a significant difference when compared to Group A; b represents a significant difference when compared to Group B; c represents a significant difference when compared to Group C; d represents a significant difference when compared to Group D.

#### 3.4.2 Hormonal assay

As shown in Table 7, the hormonal profiles showed significantly reduced GnRH, FSH, and total testosterone levels in Group B compared to the control. Group C presented the highest GnRH, FSH, and testosterone concentrations, significantly greater than Group B. Group D results showed intermediate levels of GnRH, FSH, and testosterone, significantly different from both Groups B and C. Group E also produced moderate hormone levels, which were significantly different from Group C and lower than Group D. LH levels were less variable across groups, with no significant differences except a slight reduction in Groups B and E.

**TABLE 7:** Hormonal changes among groups.

Groups	GnRH	FSH	LH	TT
A	1.70 ± 0.14	6.75 ± 0.07	3.05 ± 0.21	2.95 ± 0.07
B	1.00 ± 0.14^a^	2.30 ± 1.98a	2.15 ± 0.64	1.20 ± 0.00^a^
C	1.95 ± 0.21^b^	6.85 ± 1.06b	2.90 ± 0.28	3.20 ± 0.42^b^
D	1.55 ± 0.70^bc^	4.95 ± 0.35	2.65 ± 0.07	2.15 ± 0.21^c^
E	1.40 ± 0.00	4.35 ± 0.50	2.05 ± 1.34	2.10 ± 0.28^c^

Values represent Mean ± SD. a represents a significant difference when compared to Group A; b represents a significant difference when compared to Group B; c represents a significant difference when compared to Group C.

#### 3.4.3 Antioxidant markers

Antioxidant markers presented in Table 8 showed that Group B had a significant reduction in catalase (16.85 u/g) and glutathione (12.80 μg/dL) compared to the control. Group C produced the highest levels of catalase (28.30 u/g) and glutathione (27.50 μg/dL), significantly greater than Group B. Group D resulted in catalase and glutathione levels that were higher than Group B but lower than Group C, while Group E showed intermediate values, significantly different from Group C.

**TABLE 8:** Antioxidant markers.

Groups	CAT (u/g)	GSH (ug/dl)
A	23.30 ± 1.34	23.15 ± 2.48
B	16.85 ± 1.20^a^	12.80 ± 3.39^a^
C	28.30 ± 2.83^b^	27.50 ± 2.12^b^
D	22.30 ± 0.14	21.50 ± 2.12
E	22.05 ± 0.64^c^	20.00 ± 0.00

Values represent Mean ± SD. a represents a significant difference when compared to Group A; b represents a significant difference when compared to Group B; c represents a significant difference when compared to Group C.

#### 3.4.4 Inflammatory cytokines

Inflammatory cytokine levels across experimental groups are presented in Table 9. Group B increased TNF-α to 35.20 pg/mg protein and significantly reduced IL-4–5.85 pg/mg protein compared to the control. Group C showed TNF-α and IL-4 levels of 28.40 and 10.15 pg/mg protein, respectively, with IL-4 significantly higher than Group B. Group E showed TNF-α of 25.85 pg/mg and IL-4 of 8.35 pg/mg, with IL-4 significantly lower than Group C.

**TABLE 9:** Inflammatory cytokines.

Groups	TNF-α (pg/mg protein)	IL-4 (pg/mg protein)
A	25.95 ± 3.47	11.40 ± 1.41
B	35.20 ± 8.63	5.85 ± 0.64^a^
C	28.40 ± 7.07	10.15 ± 0.07^b^
D	28.45 ± 5.59	9.40 ± 0.00
E	25.85 ± 1.91	8.35 ± 1.34

Values represent Mean ± SD. a represents a significant difference when compared to Group A; b represents a significant difference when compared to Group B; c represents a significant difference when compared to Group C.

#### 3.4.5 Steroidogenic enzymes

Steroidogenic enzyme activities of StAR, 3β-HSD, and 17β-HSD were significantly reduced in Group B compared to the control. Group C showed the highest enzyme activities, significantly greater than Group B. Group D resulted in intermediate enzyme activities significantly different from Groups B and C, while Group E also showed partial restoration of enzyme levels significantly different from Groups A, C, and D (Table 10).

**TABLE 10:** Steroidogenic enzymes.

Groups	3β-HSD (U/mg protein)	17β-HSD (U/mg protein)	StAR (U/mg protein)
A	17.05 ± 0.21	16.10 ± 0.42	19.70 ± 0.28
B	8.00 ± 0.28^a^	9.70 ± 0.14a	11.03 ± 0.24^a^
C	18.60 ± 0.42^ab^	18.15 ± 0.07ab	22.50 ± 0.55^ab^
D	11.00 ± 0.42^abc^	11.95 ± 0.78abc	13.67 ± 0.47^abc^
E	10.35 ± 0.35^abc^	10.65 ± 0.07ac	11.94 ± 0.38^acd^

Values represent Mean ± SD. a represents a significant difference when compared to Group A; b represents a significant difference when compared to Group B; c represents a significant difference when compared to Group C; d represents a significant difference when compared to Group D.

Caspase-3 and caspase-9 activities are presented in Table 11. Group B showed a marked elevation in both caspase-3 and caspase-9 activities compared with the control group, indicating enhanced apoptotic signalling. In contrast, Group C recorded significantly lower activities relative to Group B. Groups D and E were significantly reduced compared with Group B but remained significantly higher than Groups A and C.

**TABLE 11:** Caspase-3-9 activities.

Groups	Cas-3 (pg/mg prt)	Cas-9 (pg/mg prt)
A	94.56 ± 1.77	115.20 ± 1.84
B	223.60 ± 0.92^a^	302.80 ± 3.61^a^
C	84.40 ± 3.25^b^	103.00 ± 2.19^b^
D	160.00 ± 4.24^abc^	225.90 ± 0.85^abc^
E	197.00 ± 4.24^abcd^	267.40 ± 6.72^abcd^

Values represent Mean ± SD. a represents a significant difference when compared to Group A; b represents a significant difference when compared to Group B; c represents a significant difference when compared to Group C; d represents a significant difference when compared to Group D.

### 3.5 Histological findings

**FIGURE 2:**
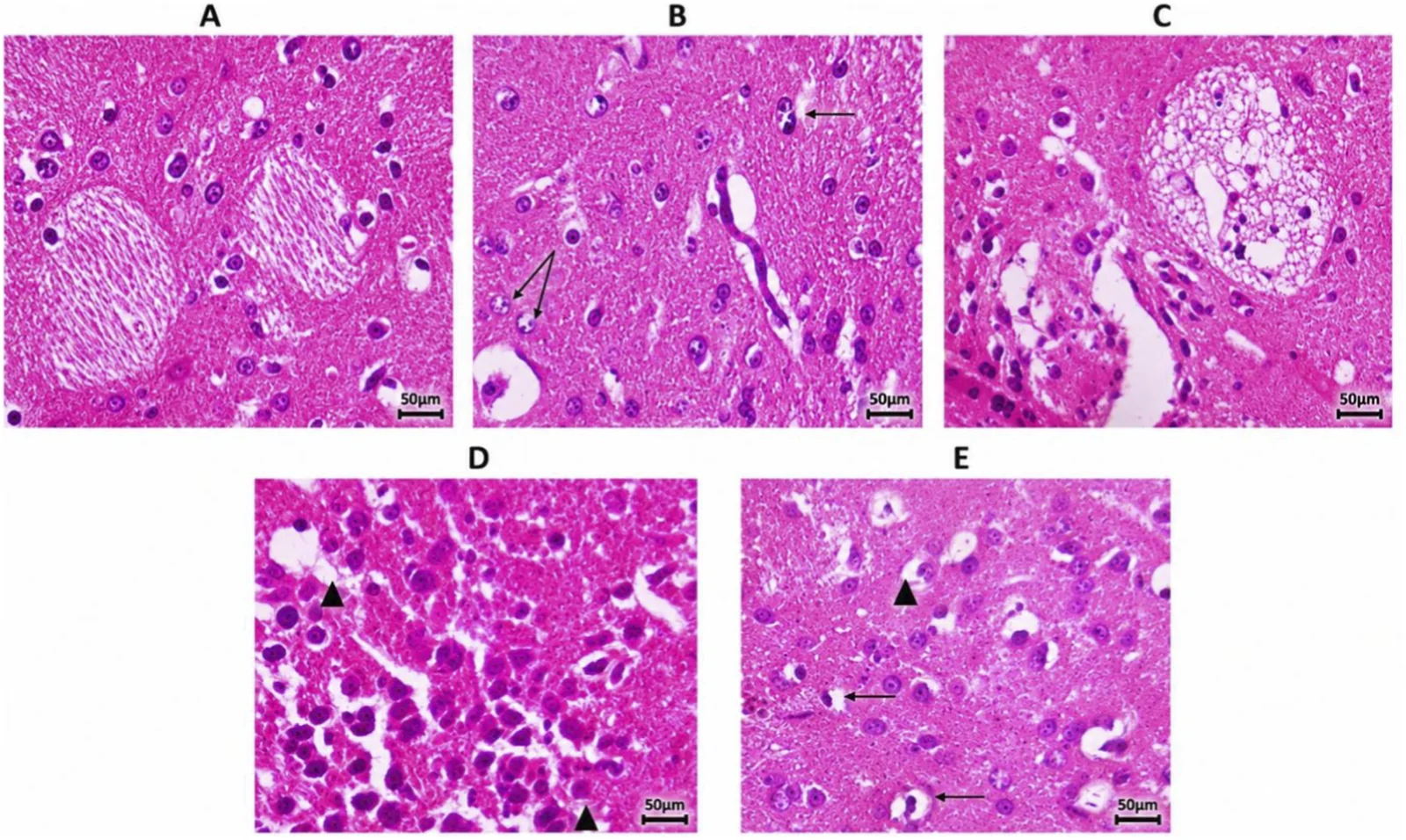
Representative photomicrographs of the hypothalamus from the experimental groups: **(A)** Control, **(B)** Heavy Metal + Recovery, **(C)** ALA Only, **(D)** Post-Treatment, and **(E)** Pre-Treatment. H&E stain; ×400. Scale bar = 50 μm. Arrow, neuronal degeneration; arrowhead, cytoplasmic vacuolation.

**FIGURE 3:**
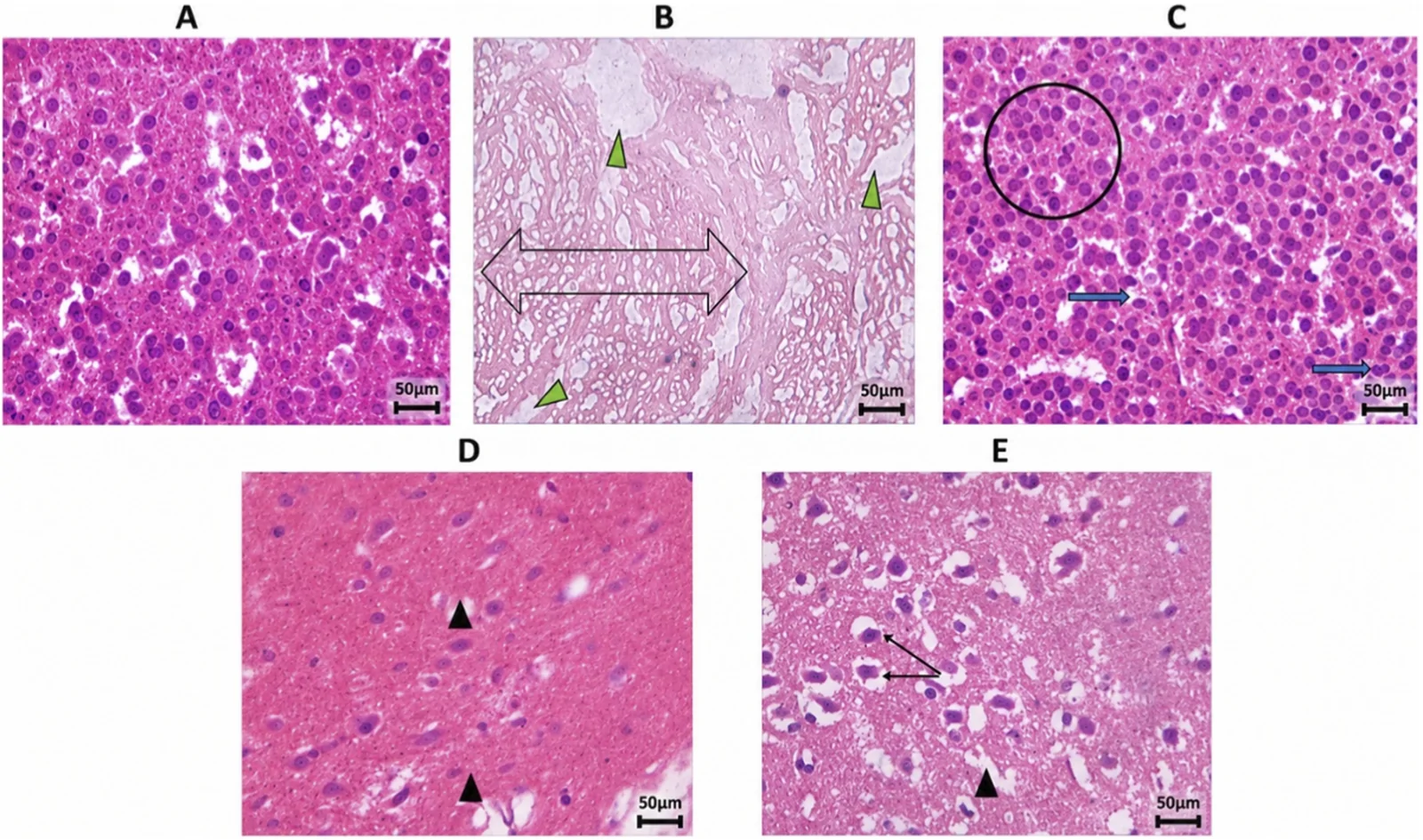
Representative photomicrographs of the pituitary gland from the experimental groups: **(A)** Control, **(B)** Heavy Metal + Recovery, **(C)** ALA Only, **(D)** Post-Treatment, and **(E)** Pre- Treatment. H&E stain; ×400. Scale bar = 50 μm. Hollow arrow, reduced cellular density; green arrowhead, intercellular oedema; dark circle, densely packed endocrine cells; blue arrow, basophils; arrow, cellular degeneration; black arrowhead, vacuolation.

**FIGURE 4:**
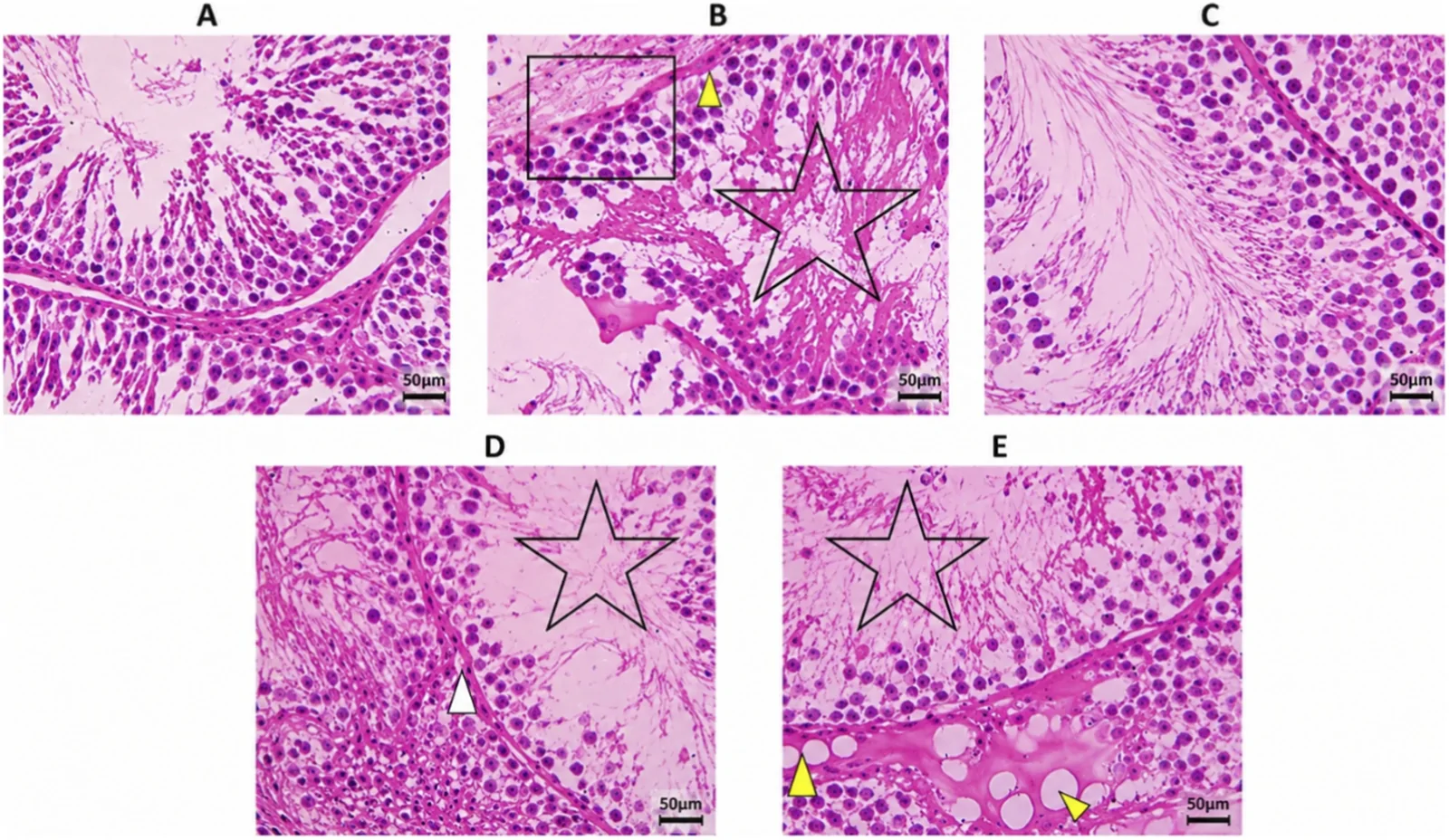
Representative photomicrographs of the testes from the experimental groups: **(A)** Control, **(B)** Heavy Metal + Recovery, **(C)** ALA Only, **(D)** Post-Treatment, and **(E)** Pre-Treatment. H&E stain; ×400. Scale bar = 50 μm. Black block, degeneration of seminiferous tubules; hollow star, loss of germinal epithelium; white arrowhead, cytoplasmic vacuolation; yellow arrowhead, interstitial tissue changes.

Representative photomicrographs of the hypothalamus, pituitary gland, and testes are presented in Figures 2–4, respectively.

#### 3.5.1 Hypothalamus

The control group (Group A) showed normal hypothalamic architecture characterised by intact neurons with well-defined cell bodies, prominent nuclei, and minimal intercellular spaces. In contrast, exposure to heavy metal-contaminated water (Group B) induced marked histopathological alterations, including widespread neuronal degeneration, cytoplasmic vacuolation, ischemic changes, and pyknosis. The ALA-only group (Group C) maintained normal cytoarchitecture comparable to the control. Post-treatment with alpha-lipoic acid (Group D) resulted in moderate improvement, with reduced vacuolation and neuronal degeneration, although mild inflammatory cell infiltration and residual vacuoles persisted. Pre-treatment with ALA (Group E) offered limited protection, showing persistent neuronal degeneration, vacuolation, and gliosis similar to the heavy metal-exposed group.

#### 3.5.2 Pituitary gland

Control animals displayed typical pituitary histology with densely packed endocrine cells, a predominance of basophils, and sparse acidophils within a well-vascularised stroma. Heavy metal exposure (Group B) caused significant disruption, evidenced by reduced cellular density, intercellular oedema, marked vacuolation, and cellular degeneration. The ALA-only group (Group C) exhibited normal pituitary morphology with densely arranged endocrine cells. Post-exposure ALA treatment (Group D) partially restored cellular density and reduced vacuolation, though mild oedema and scattered degenerated cells remained. Pre-treatment (Group E) showed only marginal improvement, with persistent vacuolar spaces, reduced cell density, and focal areas of degeneration.

#### 3.5.3 Testes

In the control group, the seminiferous tubules exhibited normal architecture with intact germinal epithelium comprising all stages of spermatogenesis, active spermiogenesis, and regular tubular diameter. Leydig cells in the interstitium appeared normal. Heavy metal exposure (Group B) produced severe testicular damage, including degeneration and sloughing of germ cells, loss of germinal epithelium, tubular atrophy, cytoplasmic vacuolation of Sertoli cells, interstitial oedema, and reduced Leydig cell population. The ALA-only group (Group C) showed well-preserved seminiferous tubule morphology and active spermatogenesis indistinguishable from the control. Post-treatment with ALA (Group D) significantly attenuated the damage, resulting in improved germ cell organisation, reduced vacuolation, and partial restoration of spermatogenesis, although mild tubular degeneration and interstitial widening persisted. Pre-treatment (Group E) provided comparatively less protection, with noticeable germ cell depletion, interstitial oedema, and incomplete recovery of spermatogenic activity.

### 3.6 Histopathological evaluation

Histopathological evaluations, including the Johnsen score and histological scoring, are presented in Table 12. Group B showed marked reductions in the Johnsen score (5.00) and higher histological scores in the hypothalamus, pituitary and testes compared to the control. Group C maintained normal tissue architecture, with the highest Johnsen score and the lowest histological scores, which were significantly different from those of Group B. Group D showed moderate improvements in Johnsen and histological scores relative to Group B. Group E showed partial restoration, with Johnsen and histological scores intermediate between Groups B and D, significantly different from Group C.

**TABLE 12:** Johnsen score and histological scoring results.

Groups	Johnsen testicular score	Histological scoring		
​	​	Hypothalamus	Pituitary gland	Testes
A	9.00	0.71 ± 0.49	0.50 ± 0.53	0.50 ± 0.55
B	5.00^a^	2.13 ± 0.64^a^	1.86 ± 0.38^a^	2.00 ± 0.63^a^
C	9.50^b^	0.43 ± 0.54^b^	0.50 ± 0.53^b^	0.33 ± 0.52^b^
D	6.00	1.71 ± 0.76	1.50 ± 0.53	1.57 ± 0.53
E	5.50^c^	1.86 ± 0.69^c^	1.50 ± 0.53	1.50 ± 0.55

Values represent Mean (for Johnsen score) and Mean ± SD (for histological scoring). a represents a significant difference when compared to Group A; b represents a significant difference when compared to Group B; c represents a significant difference when compared to Group C.

### 3.7 Testicular histomorphometry

#### 3.7.1 Testicular Component Area Fractions (TCAF)

Testicular component area fractions are summarised in Table 13. Heavy-metal exposure in Group B resulted in a marked reduction in seminiferous epithelium area compared to the control and a corresponding increase in connective tissue, lumen, and interstitial space. Group C showed the highest seminiferous epithelium proportion and significantly lower connective tissue, lumen, and interstitial space compared to Group B. Post-treatment with ALA in Group D produced seminiferous epithelium and connective tissue, significantly different from Groups A and C. Group E yielded seminiferous epithelium, connective tissue and interstitial space, also significantly different from Groups A and C.

**TABLE 13:** Testicular component area fractions (%).

Groups	A	B	C	D	E
TCAF (%)					
Seminiferous epithelium	60.33 ± 1.53	46.67 ± 1.53^a^	66.00 ± 2.00^b^	48.67 ± 2.52^ac^	47.33 ± 3.22^ac^
Connective tissue	9.83 ± 0.98	14.33 ± 0.82^a^	8.33 ± 0.82^b^	12.33 ± 1.21^abc^	13.20 ± 0.84^ac^
Lumen	4.75 ± 0.50	6.75 ± 0.50^a^	3.50 ± 0.58^ab^	5.50 ± 0.58^bc^	5.75 ± 0.50^c^
Interstitial space	4.40 ± 0.55	8.80 ± 0.84^a^	3.40 ± 0.55^b^	6.80 ± 0.84^abc^	8.20 ± 0.84^ac^

Values represent Mean ± SDa, represents a significant difference when compared to Group A; b represents a significant difference when compared to Group B; c represents a significant difference when compared to Group C.

#### 3.7.2 Seminiferous Tubule Morphometry (STM)

Seminiferous tubule morphometry is presented in Table 14. Group B had reduced tubule area, tubule diameter, and epithelium height, while lumen diameter increased compared with the control and Group C, which showed the highest tubule area, largest tubule diameter, and greatest epithelium height, with significantly smaller lumen diameter. Groups D and E showed significant restoration, with increased tubule area, tubule diameter, and epithelium height relative to Groups A, B and C, respectively.

**TABLE 14:** Seminiferous tubule morphometry.

Groups	A	B	C	D	E
STM (µm)					
Tubule Area	65,614 ± 430.5	55,835 ± 1001^a^	70,788 ± 350.50^ab^	58,869 ± 514.10^abc^	58,008 ± 755.50^abc^
Tubule diameter	248.50 ± 8.20	235.00 ± 7.59^a^	259.30 ± 6.83^ab^	242.00 ± 9.14^abc^	244.10 ± 8.42^abc^
Epithelium height	77.50 ± 1.29	62.25 ± 1.50^a^	88.00 ± 0.82^ab^	69.25 ± 1.50^abc^	67.50 ± 1.73^abc^
Lumen diameter	93.50 ± 2.38	110.50 ± 1.29^a^	83.25 ± 1.71^ab^	102.50 ± 2.38^abc^	108.80 ± 1.26^acd^

Values represent Mean ± SD. a represents a significant difference when compared to Group A; b represents a significant difference when compared to Group B; c represents a significant difference when compared to Group C; d represents a significant difference when compared to Group D.

#### 3.7.3 Germinal Epithelium Cell Quantification (GECQ)

Germinal epithelium cell quantification is shown in Table 15. Heavy-metal exposure in Group B resulted in significantly lower counts of Sertoli cells, spermatogonia, spermatocytes, round spermatids, and elongated spermatids compared with the control. Group C had the highest values across all cell types, with significant increases over Groups A and B. In the post-treatment group, i.e., Group D, cell counts were significantly higher than in Group B but remained significantly lower than those of Groups A and C, indicating partial restoration. Group E produced a similar pattern, with values significantly different from Groups A and C in most parameters.

**TABLE 15:** Germinal epithelium cell quantification.

Groups	A	B	C	D	E
GECQ					
Sertoli	20.20 ± 1.30	15.80 ± 0.84^a^	24.20 ± 0.84^ab^	17.80 ± 0.84^abc^	16.60 ± 0.55^ac^
Spermatogonia	64.60 ± 2.07	54.40 ± 1.14^a^	69.40 ± 1.14^ab^	57.20 ± 1.64^abc^	55.60 ± 1.14^ac^
Spermatocytes	63.60 ± 1.95	51.80 ± 1.30^a^	70.80 ± 1.48^ab^	58.00 ± 1.58^abc^	54.00 ± 0.71^ac^
Round spermatids	94.83 ± 2.48	61.50 ± 2.26^a^	101.00 ± 2.10^ab^	79.50 ± 1.52^abc^	76.33 ± 1.03^abc^
Elongated spermatids	90.80 ± 1.30	63.00 ± 1.58^a^	100.40 ± 1.14^ab^	71.60 ± 2.70^abc^	68.00 ± 2.12^abcd^

Values represent Mean ± SD. a represents a significant difference when compared to Group A; b represents a significant difference when compared to Group B; c represents a significant difference when compared to Group C; d represents a significant difference when compared to Group D.

#### 3.7.4 Leydig Cell Morphometry (LCM)

Leydig cell morphometry is presented in Table 16. Group B produced significantly reduced Leydig cell diameter and volume relative to Group A. Group C showed the highest values for both parameters, with significant increases compared to Groups A and B. Post-treatment resulted in improvements, remaining significantly lower than Groups A and C but higher than Group B. Pre-treatment (Group E) also showed partial improvement, with values significantly different from Groups A, C, and D in most comparisons.

**TABLE 16:** Leydig cell morphometry.

Groups	A	B	C	D	E
LCM					
Leydig diameter (µm)	4.48 ± 0.13	3.88 ± 0.10^a^	4.88 ± 0.10^ab^	4.15 ± 0.13^abc^	4.00 ± 0.08^ac^
Leydig volume (µm^3^)	73.50 ± 2.89	49.25 ± 1.50^a^	86.75 ± 0.96^ab^	58.25 ± 1.50^abc^	54.25 ± 0.96^abcd^

Values represent Mean ± SD. a represents a significant difference when compared to Group A; b represents a significant difference when compared to Group B; c represents a significant difference when compared to Group C; d represents a significant difference when compared to Group D.

## 4 Discussion

To the best of our knowledge, few studies have examined the effects of naturally contaminated borehole water on the complete hypothalamus–pituitary–gonadal (HPG) axis and the timing-dependent protective effects of alpha-lipoic acid. The present work employed an environmentally realistic exposure model through naturally contaminated drinking water collected from a mining-impacted community in Ebonyi State, Nigeria, containing Pb (2.4 mg/L), Cd (1.7 mg/L), As (1.2 mg/L), and Hg (0.8 mg/L), concentrations that closely mirror the exposure scenario faced by rural populations in mining regions of sub-Saharan Africa. This study provides clear evidence on the timing-dependent ameliorative effects of ALA, demonstrating that post-exposure administration confers significantly greater protection than pre-exposure prophylaxis.

The study design included a recovery-only group (Group B: 28 days contaminated water followed by 14 days distilled water only). The post-treatment group (Group D) showed statistically superior improvements in sperm motility, testosterone levels, antioxidant status, steroidogenic enzyme activities, and Johnsen’s score compared with the recovery-only group. These findings indicate that the observed benefits were primarily attributable to ALA rather than spontaneous recovery alone.

Borehole water analysis from Alike, Ikwo LGA, showed Pb, Cd, As, and Hg concentrations exceeding WHO limits by 6.1-, 18-, 5-, and 6.7-fold, respectively. These findings align with earlier reports from the Royal Salt mining area in Ebonyi State (Okafor et al., 2024; Okafor, 2025), highlighting the public health significance of this exposure model, which better reflects real-world scenarios than traditional single-metal or high-dose mixtures.

The observed weight loss and decreased brain/testicular weights in the heavy-metal-exposed group reflect the systemic effects of heavy metals, which impair nutrient absorption and promote protein catabolism (Deore et al., 2021; Uchewa et al., 2025). Similar reductions have been seen in rats exposed to Pb at 15 mg/kg/day for 10 days (Ayoka et al., 2016). Our mixed-metal model, despite lower individual concentrations, suggests additive toxicity, as Cd and As amplify Pb’s disruptive effects (Liu et al., 2023; Yoo et al., 2021). ALA post-treatment partially restored weights, indicating its role in countering metal-induced metabolic issues (Petronilho et al., 2016), but our 25 mg/kg regimen may be suboptimal compared to higher doses (100–200 mg/kg) used in other studies (Petronilho et al., 2016). Pre-treatment with ALA provided even less protection, likely due to its transient tissue accumulation (Shay et al., 2009), highlighting the need for sustained ALA administration in chronic exposures to combat progressive toxicity.

Semen parameters (motility, count, and normal morphology) were markedly impaired, with increased pinhead and midpiece defects. These changes reflect heavy metal-induced oxidative damage to sperm membranes and DNA (Esomchi et al., 2025), consistent with epidemiological data from mining communities (Inhorn et al., 2008; Abilash and Sridharan, 2024) and experimental mixture studies (Oztas et al., 2025; Marić et al., 2025). ALA post-treatment significantly improved sperm quality compared with both the heavy metal-exposed and recovery-only groups, highlighting its protective effect during active injury (Naderi et al., 2023). This may stem from chronic, low-level mixed exposure promoting deeper bioaccumulation in the seminiferous tubules, which ALA chelates less efficiently than acute single-metal exposures.

At the hormonal level, contaminated water suppressed GnRH, FSH, and testosterone, confirming disruption across the entire HPG axis (Lafuente, 2013). The greater hormonal recovery observed in the post-treatment group highlights ALA’s neuroprotective and steroidogenic-supportive actions, likely mediated through Nrf2 activation and preservation of hypothalamic and Leydig cell function (Famurewa et al., 2023; Rachamalla et al., 2022). This extends previous findings from single-metal models to a real-world multi-metal exposure scenario.

In Group B, steroidogenic enzyme downregulation suggests that heavy metals inhibit cholesterol transport and enzymatic sulfhydryls, as seen in single-metal studies (Deore et al., 2021). ALA’s intermediate upregulation helps regenerate GSH for enzyme protection, but its effectiveness is reduced compared to single-Pb models with 50–100 mg/kg (Famurewa et al., 2023), likely due to chronic exposure causing irreversible Leydig cell apoptosis (El-Maraghy and Nassar, 2011). Differences may also arise from exposure routes: drinking water leads to gradual absorption and deposition, while intraperitoneal injections cause higher peak toxicity but shorter duration.

Depletion of catalase and reduced glutathione, elevated TNF-α, and increased caspase-3/9 activity confirmed oxidative stress, inflammation, and apoptosis as central mechanisms (Famurewa et al., 2023; Uchewa et al., 2025; Li et al., 2025). ALA effectively replenished antioxidant defences and reduced apoptotic signalling when administered post-exposure, consistent with its documented ability to interrupt ongoing oxidative damage (Famurewa et al., 2023; Li et al., 2025).

Histomorphometric changes, such as reduced seminiferous epithelium, tubule diameter, germ cell counts, and Leydig cell volume, align with Johnsen scores that indicate arrested spermatogenesis and tubular atrophy due to environmental exposure (Deore et al., 2021). These structural alterations were substantially attenuated by post-exposure ALA, demonstrating protection of both spermatogenic and steroidogenic compartments. Treatment showed only marginal benefits, possibly due to insufficient ALA bioavailability to address bioaccumulated metals in Sertoli tight junctions (Naderi et al., 2023). However, persistent interstitial expansion suggests incomplete resolution of chronic inflammation and vascular leakage, differing from acute models where higher ALA doses fully normalise architecture (Li et al., 2025; Sun et al., 2025).

Limitations of the study include the relatively low dose of ALA (25 mg/kg) and the limited panel of oxidative stress markers measured. Higher doses and/or combination therapies may be required for full restoration against such a complex metal mixture. Future studies should incorporate additional mechanistic markers (e.g., Nrf2, NF-κB, Bax/Bcl-2) and a broader range of oxidative stress indices (MDA, SOD, GPx).

## 5 Conclusion

Chronic exposure to naturally heavy metal-contaminated borehole water from a mining area in Ebonyi State, Nigeria, causes profound multi-level disruption of the hypothalamus–pituitary–gonadal (HPG) axis in adult male Wistar rats. Alpha-lipoic acid (25 mg/kg) provided significant but partial protection, with post-exposure treatment being clearly more effective than pre-exposure. However, both regimens showed limited success, as the heavy metal mixture exceeding WHO limits by 5–18-fold overwhelmed ALA’s capacity at the dose used. Chronic bioaccumulation in lipophilic tissues such as the testes and hypothalamus likely outpaced ALA’s chelation and antioxidant effects, preventing full restoration. These findings highlight the serious reproductive health risks in mining communities and the need for higher ALA doses, combination therapies, and urgent water remediation.

## Data Availability

The raw data supporting the conclusions of this article will be made available by the authors, without undue reservation.
