# Correction: Chronic exposure to mining-contaminated borehole water impairs the entire male reproductive axis: partial mitigation by alpha-lipoic acid in Wistar rats

**DOI:** 10.3389/ftox.2026.1988528

**Published:** 2026-09-17

**Authors:** Festus Toochukwu Okposhi, Collins Nduka Esomchi, Kenneth Tochukwu Onwe, Leslie Ogechi Onumajuru, Ernest Maduabuchi Omeje, Favour Chioma Onwumere, Precious Chidiogo Ugwu, Goodness Emmanuella Onukogu, Jennifer Obianuju Olisaeke, Chinwendu Princess Victor, Chiemerie Promise Omeh, Bianca Adanna Onele

**Affiliations:** 1 Department of Anatomy, Faculty of Basic Medical Sciences, Alex Ekwueme Federal University Ndufu Alike, Ikwo, Ebonyi State, Nigeria; 2 Department of Human Anatomy, Faculty of Basic Medical Sciences, State University of Medical and Applied Sciences, Igbo Eno, Enugu State, Nigeria

**Keywords:** alpha-lipoic acid, borehole water, heavy metal contamination, HPG axis, male reproductive toxicity, oxidative stress

There was a mistake in the captions of Figures 2, 3, and 4 as published. The annotation keys for the hypothalamic, pituitary, and testicular photomicrographs were omitted. In addition, a production comment was incorporated into the published caption of Figure 4. The figures themselves are unchanged. The corrected captions appear below.

There was a mistake in the caption of Figure 2 as published. The annotation keys were omitted. The corrected caption of **Figure 2** appears below.

“Figure 2 | Representative photomicrographs of the hypothalamus from the experimental groups: (A) Control, (B) Heavy Metal + Recovery, (C) ALA Only, (D) Post-Treatment, and (E) Pre-Treatment. H&E stain; ×400. Scale bar = 50 μm. Arrow, neuronal degeneration; arrowhead, cytoplasmic vacuolation.”

There was a mistake in the caption of Figure 3 as published. The annotation keys were omitted. The corrected caption of **Figure 3** appears below.

“Figure 3 | Representative photomicrographs of the pituitary gland from the experimental groups: (A) Control, (B) Heavy Metal + Recovery, (C) ALA Only, (D) Post-Treatment, and (E) Pre-Treatment. H&E stain; ×400. Scale bar = 50 μm. Hollow arrow, reduced cellular density; green arrowhead, intercellular oedema; dark circle, densely packed endocrine cells; blue arrow, basophils; arrow, cellular degeneration; black arrowhead, vacuolation.”

There was a mistake in the caption of Figure 4 as published. The annotation keys were omitted, and a production comment was added to the caption. The corrected caption of **Figure 4** appears below.

“Figure 4 | Representative photomicrographs of the testes from the experimental groups: (A) Control, (B) Heavy Metal + Recovery, (C) ALA Only, (D) Post-Treatment, and (E) Pre-Treatment. H&E stain; ×400. Scale bar = 50 μm. Black block, degeneration of seminiferous tubules; hollow star, loss of germinal epithelium; white arrowhead, cytoplasmic vacuolation; yellow arrowhead, interstitial tissue changes.”

The original version of this article has been updated.

